# Multi-dimensional feature recognition model based on capsule network for ubiquitination site prediction

**DOI:** 10.7717/peerj.14427

**Published:** 2022-12-06

**Authors:** Weimin Li, Jie Wang, Yin Luo, Tsigabu Teame Bezabih

**Affiliations:** 1 School of Computer Engineering and Science, Shanghai University, Shanghai, China; 2 School of Life Sciences, East China Normal University, Shanghai, China

**Keywords:** Ubiquitination site, Capsule network, Feature recognition, Channel attention

## Abstract

Ubiquitination is an important post-translational modification of proteins that regulates many cellular activities. Traditional experimental methods for identification are costly and time-consuming, so many researchers have proposed computational methods for ubiquitination site prediction in recent years. However, traditional machine learning methods focus on feature engineering and are not suitable for large-scale proteomic data. In addition, deep learning methods are mostly based on convolutional neural networks and fuse multiple coding approaches to achieve classification prediction. This cannot effectively identify potential fine-grained features of the input data and has limitations in the representation of dependencies between low-level features and high-level features. A multi-dimensional feature recognition model based on a capsule network (MDCapsUbi) was proposed to predict protein ubiquitination sites. The proposed module consisting of convolution operations and channel attention was used to recognize coarse-grained features in the sequence dimension and the feature map dimension. The capsule network module consisting of capsule vectors was used to identify fine-grained features and classify ubiquitinated sites. With ten-fold cross-validation, the MDCapsUbi achieved 91.82% accuracy, 91.39% sensitivity, 92.24% specificity, 0.837 MCC, 0.918 F-Score and 0.97 AUC. Experimental results indicated that the proposed method outperformed other ubiquitination site prediction technologies.

## Introduction

Ubiquitination is one of the most important post-translational modifications (PTMs) processes of proteins. Protein ubiquitination is a process in which ubiquitin conjugates to the substrate protein under the catalysis of E1 activation enzymes, E2 conjugation enzymes and E3 ligation enzymes (Herrmann, Lerman & Lerman, 2007). Studies have shown that the ubiquitin-proteasome pathway plays a significant role in the regulation of many biological processes, such as DNA repair, cell apoptosis and cell proliferation (Tu et al., 2012). In addition, ubiquitination is not only associated with inflammation, cancers and neurodegenerative diseases, but also plays a role in the onset of autoimmunity and muscle dystrophies (Hoeller, Hecker & Dikic, 2006; Popovic, Vucic & Dikic, 2014). Effective prediction of ubiquitinated sites is the key to understanding the mechanism of ubiquitin modification. Traditional experimental methods for predicting ubiquitinated sites are costly and time-consuming, so it is necessary to develop efficient and accurate computational methods. In this article, the prediction of ubiquitinated sites is regarded as a dichotomous classification problem, in which we can determine whether a site belongs to a ubiquitinated site or a non-ubiquitinated site.

There are some computational methods based on machine learning which have been used to predict ubiquitinated sites. These methods can generally be divided into three categories: (1) Some approaches focus on developing or combining coding methods to improve the feature representation capability of the model. Tung & Ho (2008) proposed an IPMA algorithm and combined the algorithm with support vector machines (SVM) to design the UbiPred. Chen et al. (2011) proposed the CKSAAP encoding method and developed a predictor called CKSAAP_UbSite. Based on the CKSAAP_UbSite, hCKSAAP_UbSite (Chen et al., 2013b) was constructed by combining three other encoding methods. iUbiq-Lys (Qiu et al., 2015) based on SVM combined the sequence evolutionary information (PseAAC). (2) Some remove redundant features to improve the performance of the classifier by using feature selection algorithms. Chen et al. (2013a) used the information gain (IG) algorithm to integrate the information of key amino acid residues and key positions. The principle of maximum relevance and minimum redundancy (mRMR) was used to improve the performance of the prediction engine (Cai et al., 2012). Both UbiSitePred (Cui et al., 2019) and Ubisite-Xgboost (Liu et al., 2021b) used LASSO to remove redundant information. (3) Others have been proposed to develop efficient classifiers. Lee et al. (2011) developed a predictor using a radial basis function network. Huang et al. (2016) constructed a two-layer SVM model called UbiSite. Zhao et al. (2011) designed an ensemble random forest classifier using feature vectors and extracted four features from protein sequences. RUBI (Walsh, Di Domenico & Tosatto, 2014) was constructed with an iterative approach. Efficient Bayesian Multivariate Classifier (EBMC) (Cai & Jiang, 2016) was combined with 531 physicochemical properties. ESA-UbiSite (Wang et al., 2017) used the ESA algorithm to screen ubiquitinated sites.

The generation of large-scale proteomics data has led to the analysis of large amount of ubiquitination data, but traditional machine learning methods are not applicable on this topic. Therefore, deep learning models for large-scale ubiquitinated protein data have also been developed, mainly in terms of combining encoding methods and using transfer learning strategies. In terms of fusing encoding methods, He et al. (2017) proposed a multimodal deep learning model, which fed sequence information, physicochemical property information and evolutionary information of proteins into three sub-networks. In addition, DeepUbi (Fu et al., 2019) extracted four different features from protein and used convolutional neural networks (CNNs) to predict. In terms of using transfer learning strategies, DeepTL-Ubi (Liu et al., 2021a) was developed for predicting ubiquitination sites of multiple species. Wang et al. (2020) combined the transfer learning with a multilayer CNN to identify plant ubiquitinated sites. It revealed the differences among three species of animals, plants and fungi based on the sequences of ubiquitination proteins.

At present, capsule network has been applied to PTMs site prediction. The CapsNet architecture proposed by Wang, Liang & Xu (2019) consisted of three one-dimensional convolutional layers and one fully connected layer, and had been used in seven PTM types. The first two traditional convolutional layers in the model were used to increase the representation ability. The extracted features were inputted into the latter two layers (PrimaryCaps and PTMCaps) for further feature abstraction. This CapsNet model only extracted sequence dimension features by convolution operation, and it was not applied to ubiquitination site prediction. Caps-Ubi proposed by Luo et al. (2022) used capsule network to predict ubiquitination sites, it also ignored two dimensions of features.

Although the existing computational methods have achieved considerable performance, the following challenges remain in how to better predict large-scale protein ubiquitination sites: (1) The machine learning models perform effectively on small-scale data, but it is necessary to develop deep learning methods due to the large-scale proteomic data. (2) Some of the currently available deep learning models improve the performance of prediction tools by merging multiple encoding methods to obtain more features of the ubiquitinated protein. However, the above methods only extract single-dimensional features under different encoding methods through convolution operation. This ignores features of other dimensions, such as the feature map dimension. (3) Most deep learning models used to predict ubiquitinated sites are based on traditional CNNs. Although CNNs have achieved excellent classification performance in this field, its internal neurons are all scalars and cannot represent the hierarchical dependence between high-level features and low-level features. Therefore, the existing methods still have some limitations.

To address the drawbacks of the existing computational models, a new deep learning model MDCapsUbi was proposed. The proposed model which encodes protein fragments as feature vectors, recognizes the features of input data in sequence dimension and feature map dimension respectively through convolution operation and channel attention mechanism. After obtaining the coarse-grained features of the above two dimensions, the capsule mapping (CapsMap) layer of the capsule network further fuses the features of the two dimensions and refines them into capsule vectors. Features are transformed in a coarse-to-fine fashion. The different capsule vectors also reflect different amino acid position relationships on protein fragments, namely motifs. The capsule classification (CapsClassify) layer is composed of a positive capsule and a negative capsule. The mapping relationship between the low-level features represented by the CapsMap and the high-level features represented by the CapsClassify is determined by the dynamic routing algorithm. The routing process is more effective than the pooling operation in traditional CNNs. With the effective combination of multi-dimensional feature recognition and capsule network, MDCapsUbi achieves superior performance compared with other computational models.

## Materials & Methods

### Dataset

The large-scale protein ubiquitination dataset used in this article is collected from PLMD (Xu et al., 2017), which is the largest online lysine modification database and contains 121,742 ubiquitination sites from 25,103 proteins. To avoid interference from redundant information of homologous sequences with high similarity, CD-HIT (Huang et al., 2010) was used to clean the original dataset. First, we used the cd-hit module to remove homologous sequences with a threshold of 40% in the original dataset. Then, 60,879 protein ubiquitination sites from 17,406 proteins were obtained. The negative samples were also extracted from these protein sequences. To avoid homologous interference between the sequences in the negative samples and the positive samples, the cd-hit-2d module was used to filter the sequences in the negative samples with more than 50% similarity to the positive samples, and 320,083 non-ubiquitination sites were obtained. Following the previous study (Fu et al., 2019; Khanal et al., 2022), we selected the same number of positive and negative samples to form a balanced dataset (the number of both ubiquitinated and non-ubiquitinated sites was 60,879).

### Architecture design

**Figure 1:**
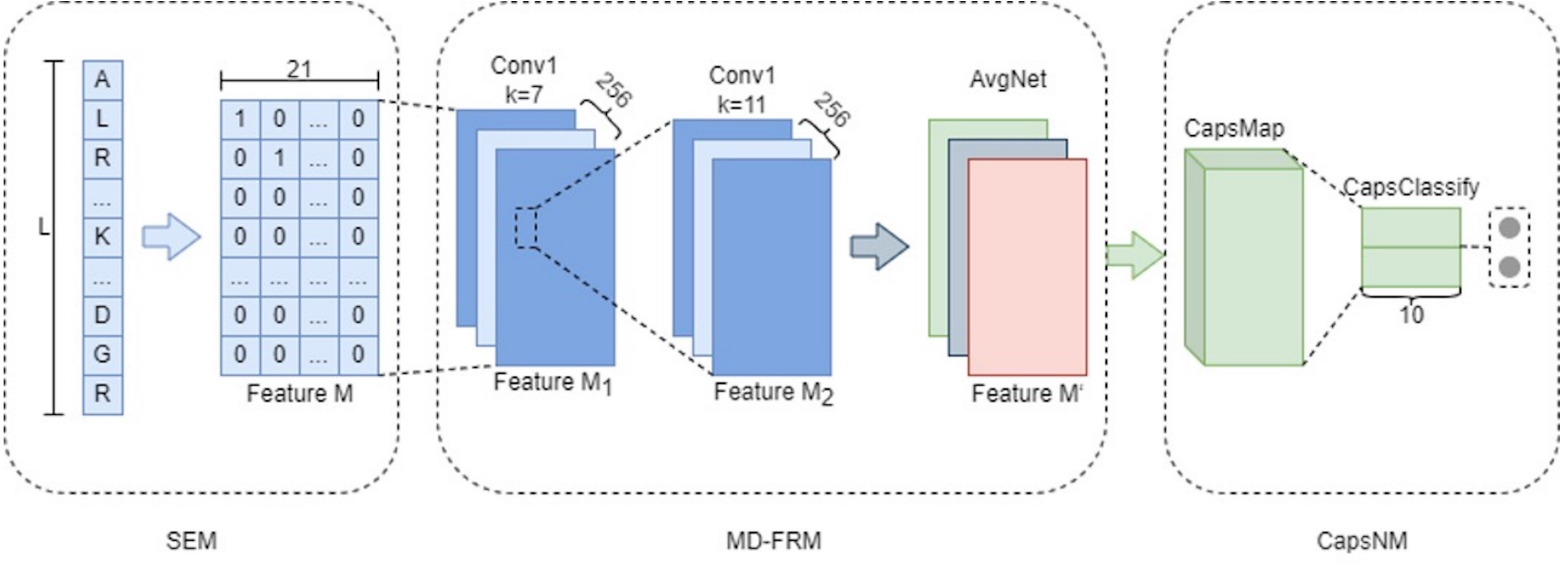
The architecture of the proposed MDCapsUbi. MDCapsUbi consists of SEM, MD-FRM and CapsNM. The length of the protein fragment in SEM is L. The dimension of feature M in SEM is 21*L. MD-FRM includes a convolutional block and channel attention. The number of channels in the first 1D convolutional layer (Conv1) is 256, and the kernel size is 7. The number of channels in the second 1D convolutional layer (Conv1) is 256, and the kernel size is 11. The channel attention is AvgNet. CapsNM consists of CapsMap and CapsClassify. The dimensions of capsules are 10.

MDCapsUbi consists of a sequence encoding module (SEM), multi-dimensional feature recognition module (MD-FRM) and capsule network module (CapsNM). SEM intercepts the raw protein sequence into protein fragments and encodes the amino acids on the protein fragments. Protein fragments are mapped as numerical vectors. MD-FRM identifies multi-dimensional hidden features by convolution operations and channel attention mechanism. CapsNM fuses and refines the features of two dimensions and realizes the classification of ubiquitinated sites and non-ubiquitinated sites. The architecture of the proposed MDCapsUbi is shown in Fig. 1.

#### Sequence Encoding Module (SEM)

To obtain the input data, *n* amino acids are extracted from each side of a ubiquitinated site to form L-long protein fragments. If the protein fragment length is less than the window length L, the corresponding position is represented by supplementary amino acid X. One-hot encoding is used to encode protein fragments. For the 20 commonly used amino acids (ACDEFGHIKLMNPQRSTVWY) and the supplementary amino acid X, each amino acid is mapped as a numeric vector with 21*1 dimension consisting of 0 and 1. For example, A is encoded as [1,0,0,0,0,0,0,0,0,0,0,0,0,0,0,0,0,0,0,0,0], and X is encoded as [0,0,0,0,0,0,0,0,0,0,0,0,0,0,0,0,0,0,0,0,1]. Eventually, a protein fragment can be represented as a 21*L feature matrix M, which is expressed as follows:

(1) \begin{eqnarray*}M& = \left[ {m}_{1},{m}_{2},{m}_{3},\ldots ,{m}_{L} \right] \end{eqnarray*}

(2) \begin{eqnarray*}L& =2\ast n+1\end{eqnarray*}

#### Multi-dimensional Feature Recognition Module (MD-FRM)

To improve the ability of the model to learn complex features, the features of protein fragments are divided into coarse-grained features and fine-grained features. This module is mainly used to identify coarse-grained features of proteins. MD-FRM recognizes sequence dimension features through convolution operations and further recognizes higher-order hidden features of feature map dimension through channel attention mechanism.

In feature recognition of sequence dimension, a convolution block is used for feature matrix *M*. The convolution block consists of two one-dimensional convolution layers and linear activation functions (ReLU). Then we obtain the feature maps *M*_1_ and *M*_2_. The operations of the convolution block are defined as follows:

(3) \begin{eqnarray*}{M}_{1}& =ReLU \left( Conv \left( M,{W}_{1},{b}_{1} \right) \right) \end{eqnarray*}

(4) \begin{eqnarray*}{M}_{2}& =ReLU \left( Conv \left( {M}_{1},{W}_{2},{b}_{2} \right) \right) \end{eqnarray*}

where *b*_1_, *b*_2_, *W*_1_ and *W*_2_ indicate the biases and weight matrices of the first convolution layer and the second convolution layer respectively. $Conv \left( . \right) $ defines the convolution operation.

In feature recognition of feature map dimension, the global average-pooling operation is used to compress feature map *M*_2_ first, and aggregate information on each channel of feature maps is obtained. The aggregated feature information is then fed into a two-layer fully connected network to obtain the weight feature, which has the same number of channels as the feature map *M*_2_. Finally, *M*_2_ is multiplied by the weight feature to obtain the final map *M*′. The relevant operations are defined as follows:

(5) \begin{eqnarray*}{F}_{avg}& =GAP \left( {M}_{2} \right) \end{eqnarray*}

(6) \begin{eqnarray*}C& =F{C}_{2} \left( F{C}_{1} \left( {F}_{avg},{W}_{3} \right) ,{W}_{4} \right) \end{eqnarray*}

(7) \begin{eqnarray*}{M}^{{}^{{^{\prime}}}}& ={M}_{2}\otimes C\end{eqnarray*}

where $GAP \left( . \right) $ defines the global average-pooling operation. *F*_*avg*_ indicates the average-pooled feature with dimension size 1*1*h (h is the number of channels in the feature map). *FC*_1_(.) and *FC*_2_(.) define fully connected networks, which use ReLU and Sigmoid as activation functions, respectively. *W*_3_ and *W*_4_ indicate the weights of the two fully connected layers respectively.

#### Capsule Network Module (CapsNM)

Compared with CNN, the capsule network is composed of capsules instead of scalar neurons. A capsule means a vector, which is a set of neurons. The capsule network in this article adopts a similar capsule network structure in (Sabour, Frosst & Hinton, 2017), consisting of the CapsMap layer and CapsClassify layer.

CapsMap can identify fine-grained features by further fusing and refining the features identified in the sequence dimension and the feature map dimension, and it converts them into capsule vectors. That means coarse-grained features are further converted into fine-grained features. This layer is similar to a one-dimensional convolution layer, but the scalar neurons are replaced by capsule vectors. There are only two capsule vectors inside the CapsClassify, one is a positive capsule and the other is a negative capsule. Their length represents the probability of ubiquitinated site and non-ubiquitinated site respectively. The dimension of capsules in CapsNM is 10D. The nonlinear mapping between CapsMap and CapsClassify is established by an iterative algorithm. This special nonlinear mapping is called dynamic routing mechanism (Sabour, Frosst & Hinton, 2017), which enables network internal parameters to be updated. The relevant operations are defined as follows:

(8) \begin{eqnarray*}{u}_{i}& =CapsConv \left( {M}^{{}^{{^{\prime}}}} \right) ,i=1,2,\ldots ,N\end{eqnarray*}

(9) \begin{eqnarray*}{\hat {u}}_{j{|}i}& ={W}_{ij}{u}_{i},j=1,2\end{eqnarray*}

(10) \begin{eqnarray*}{o}_{j}& =ROUTING \left( {\hat {u}}_{j{|}i},r,l \right) \end{eqnarray*}

where *CapsConv*(.) indicates the capsule mapping operation of CapsMap layer, *u*_*i*_ indicates *i* capsule vectors obtained. *W*_*ij*_ is a trainable weight matrix. ${\hat {u}}_{j{|}i}$ indicates the prediction vector from capsule *i* to capsule $j.ROUTING \left( {\hat {u}}_{j{|}i},r,l \right) $ is the dynamic routing algorithm and *l* denotes CapsMap layer. The hyperparameter *r* indicates that the dynamic routing algorithm iterates *r* times. The value of *r* does not have a significant effect on the model in this article, so *r* is 3 here. *o*_*j*_ indicates the output value of capsule *j*.

Since the length of the capsule vector means probability, Squash function is used as the activation function in the dynamic routing process. It can not only compress the norm of the vector to [0,1], but also retain the length information. The mathematical expression is as follows:

(11) \begin{eqnarray*}{o}_{j}= \frac{ \left\| {v}_{j} \right\| }{1+{ \left\| {v}_{j} \right\| }^{2}} \frac{{v}_{j}}{ \left\| {v}_{j} \right\| } \end{eqnarray*}

where *v*_*j*_ indicates the input vector of capsule *j*. Ultimately, the longer the length of the positive capsule, the higher the probability that the ubiquitination site is present. Therefore, we use L2 norm to calculate the length of the two capsule vectors. The margin loss function (Sabour, Frosst & Hinton, 2017) is used as the loss function of capsule network. Its mathematical formula is expressed as follows:

(12) \begin{eqnarray*}{L}_{j}={y}_{j}\cdot ReLU{ \left( \left( 1-m \right) - \left\| {o}_{j} \right\| \right) }^{2}+\lambda \left( 1-{y}_{j} \right) \cdot ReLu{ \left( \left\| {o}_{j} \right\| -m \right) }^{2}\end{eqnarray*}

where *y*_*j*_ is the predicted value of the model. The hyperparameter m and *λ* are 0.1 and 0.5 respectively.

Pooling operations in CNN can result in the loss of spatial information. The dynamic routing algorithm in the capsule network retains all the association information between the capsules in CapsMap and CapsClassify, which is more effective than the traditional average-pooling and max-pooling. The scalar neurons in traditional CNN summarize global features, so it cannot characterize the hierarchical relationship between low-level features and high-level features. In the capsule network, the capsules in CapsMap represent low-level features and the capsules in CapsClassify represent high-level features. In addition, while other neural networks require a sufficient number of samples for neuron training, the “equivariant” of the capsules (Wang, Liang & Xu, 2019) enables capsule network to learn effectively even from a small training set. “equivariant” means that the parameters of low-level capsules change with the change of perspective, while the probability of the presence of the corresponding high-level capsules remains unchanged.

### Evaluation metrics

Evaluation metrics such as accuracy (Acc), sensitivity (Sn), precision (Pre), specificity (Sp), Mathews correlation coefficient (MCC), F-Score and receiver operating characteristic curve (AUC) were used to assess the performance of MDCapsUbi. The formulas for evaluation metrics are shown as follows:

(13) \begin{eqnarray*}\text{ACC}& = \frac{TP+TN}{TP+TN+FP+FN} \end{eqnarray*}

(14) \begin{eqnarray*}\text{Sn}& = \frac{TP}{TP+FN} \end{eqnarray*}

(15) \begin{eqnarray*}\text{Sp}& = \frac{TN}{TN+FN} \end{eqnarray*}

(16) \begin{eqnarray*}\text{Pre}& = \frac{TP}{TP+FP} \end{eqnarray*}

(17) \begin{eqnarray*}\text{MCC}& = \frac{TP\times TN-FP\times FN}{\sqrt{ \left( TP+FP \right) \times \left( TP+FN \right) \times \left( TN+FP \right) \times \left( TN+FN \right) }} \end{eqnarray*}

(18) \begin{eqnarray*}\text{F-Score}& = \frac{2TP}{2TP+FP+FN} \end{eqnarray*}

where TP, TN, FP and FN are the number of true positive samples, true negative samples, false positive samples and false negative samples respectively.

AUC is the area under the receiver operating characteristic curve (ROC). The horizontal coordinate of the ROC curve indicates the false positive rate and its vertical coordinate indicates the true positive rate. As the area value of ROC curve, AUC can be used as an important indicator to measure the performance of a classifier.

### Model training

In this article, 10% of the balanced dataset was randomly selected as the independent test set, and the remaining 90% of the dataset was used as the training set. In the training process, we adopted the training method of k-fold cross verification. That was equivalent to generating k independent classifiers. The value of k was 10, and the final result was obtained by averaging the results of all classifiers. The number of iterations for training was set to 45.

We use the Adam stochastic optimization method (Kingma & Ba, 2014). The learning rate is set to 0.002. The exponential decay rates for the first-moment estimates and second-moment estimates are set to 0.9 and 0.999. The same training strategy was used for both MDCapsUbi and its variant models. The experimental platform is implemented by Tensorflow 2.4.0 and Keras 2.4.3. Model runs on Ubuntu 20.04.3 LTS system with NVIDIA GeForce RTX 2080 Ti.

## Results

### Performance of MDCapsUbi with different window sizes

**Figure 2:**
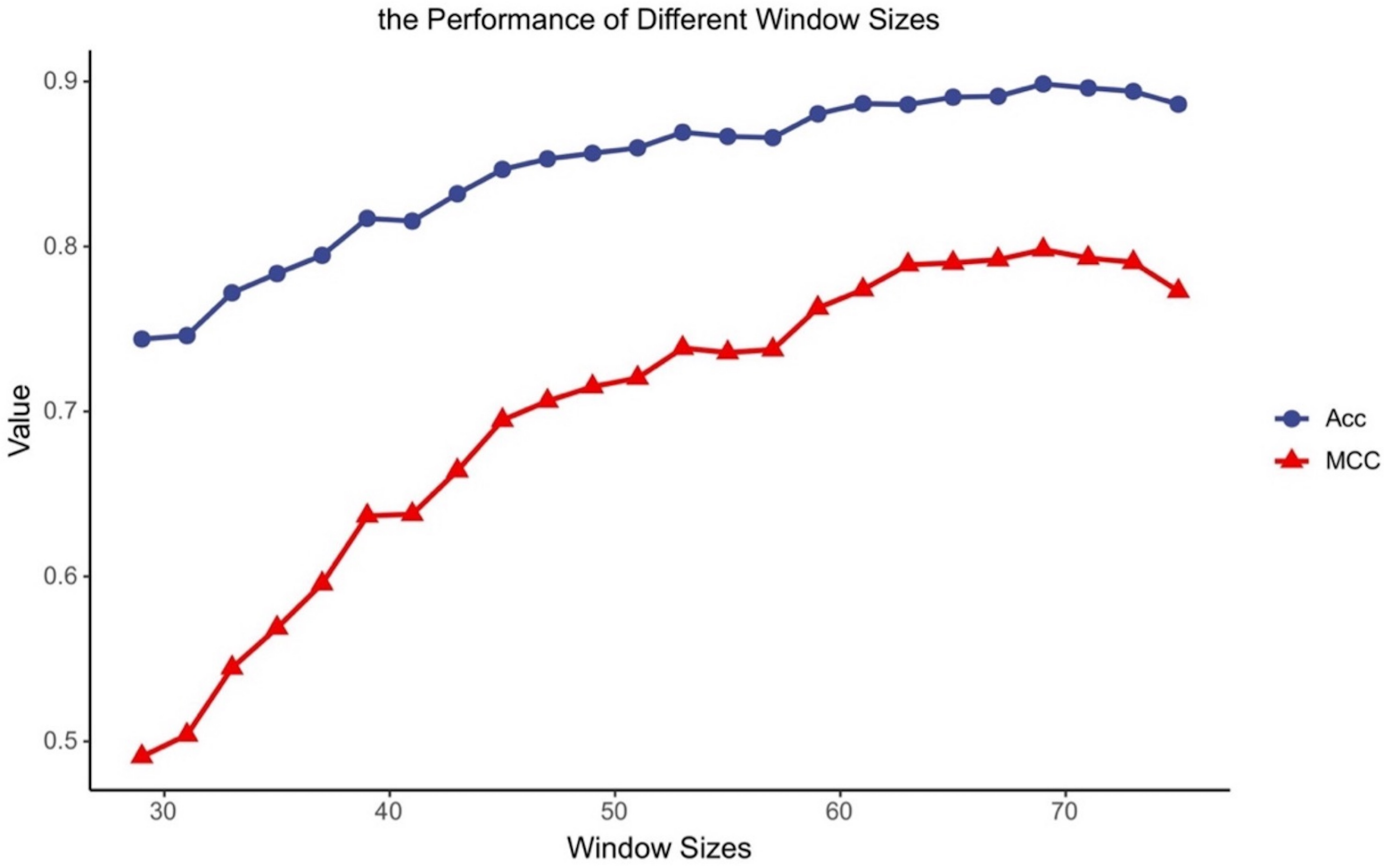
Accuracy and MCC values of MDCapsUbi with different window sizes.

Different classifiers have different structures and operating mechanisms, so they are suitable for different lengths of protein fragments. To find feasible values of the window size for MDCapsUbi, we have conducted some experiments. We increased the window size from 29 to 75 and ran 24 experiments. Then we got the accuracy and MCC values of different window sizes (Fig. 2). The performance of the model tended to increase and stabilize as the value of the window size was incremented from 29 to 69. But the model performance got worse as the window length was incremented from 71 to 75. This may be due to the fact that the sequence information recognized by the model contains more redundant information when the sequence is too long. We consider that the optimal window size L for MDCapsUbi is 69. Compared with previous studies (He et al., 2017; Fu et al., 2019; Liu et al., 2021a; Wang et al., 2020), MDCapsUbi applies to a larger window size. It also shows that the proposed model can explore more sequence information and detect deeper features of the sequences compared with other models. In addition, these experiments also verify that the long-distance features are useful for the prediction of ubiquitination sites (Qiu et al., 2015).

### Comparative experiment of MD-FRM variants

To better understand the practicability of MD-FRM, we changed the number of convolution layers and the dimension of feature recognition module. The corresponding variant models were constructed. First, two variants (MD-FR_1 and MD-FR_3) were constructed by changing the number of convolutional layers in the sequence dimension feature recognition module to study the influence of the complexity of the module. Then, we designed the single-dimension feature recognition model (SDCapsUbi), which meant that the model only recognized the features of the sequence dimension, not the features of the feature map dimension. The architecture details of MD-FRM of the models are shown in Table 1. The above models are described in detail as follows:

MD-FR_1:This model consists of SEM, MD-FRM, and CapsNM. In the MD-FRM, the number of convolutional layers used to recognize sequence dimension features is 1.

MD-FR_3:This model consists of SEM, MD-FRM, and CapsNM. In the MD-FRM, the number of convolutional layers used to recognize sequence dimension features is 3.

SDCapsUbi: This model consists of SEM, a sequence dimensional feature recognition module and CapsNM. The sequence dimension feature recognition module has the same structure as the convolution block in MDCapsUbi.

**Figure 3:**
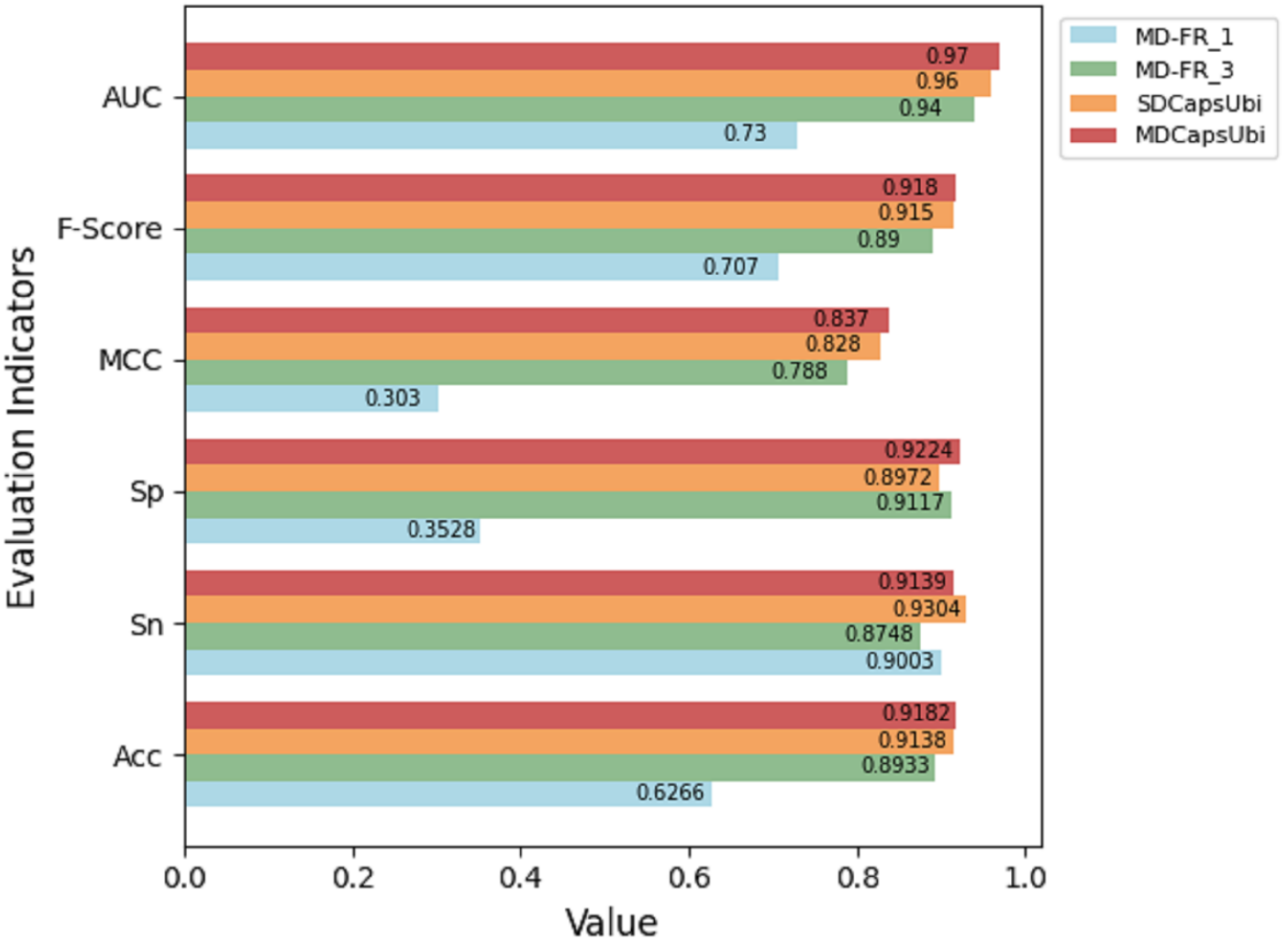
Performance comparison between MDCapsUbi and three variant models.

The test results of the above variant models were compared with MDCapsUbi. The results are shown in Fig. 3. From the figure, it can be seen that the performance of MDCapsUbi is better than that of SDCapsUbi with the same parameters. This means that the feature recognition module combining sequence dimension and feature map dimension proposed in this article is meaningful. Compared with MD-FR_1, the Acc, Sp, MCC, F-Score and AUC of MDCapsUbi are substantially improved. Compared with MD-FR_3, MDCapsUbi also shows superiority, and all evaluation indicators are higher than MD-FR_3. The above shows that for the model and application scenarios in this article, the effect of one convolution layer in the process of feature extraction is limited. On the contrary, too many convolutional layers not only increase the complexity and running time of the model, but also add redundant information, which affect the model in a bad way. The experiments also demonstrate the importance of the appropriate number of convolution layers to capture hidden features in the sequences.

**Table 1:** Architecture details of MD-FRM.

Layer name	MD-FR_1		MD-FR_3		SDCapsUbi		MDCapsUbi	
	filters	kernel_size	filters	kernel_size	filters	kernel_size	filters	kernel_size
Conv1D_1	256	7	256	7	256	7	256	7
Conv1D_2	–	–	256	11	256	11	256	11
Conv1D_3	–	–	256	11	–	–	–	–
Dropout	0.3							

### Effectiveness of channel attention

**Figure 4:**
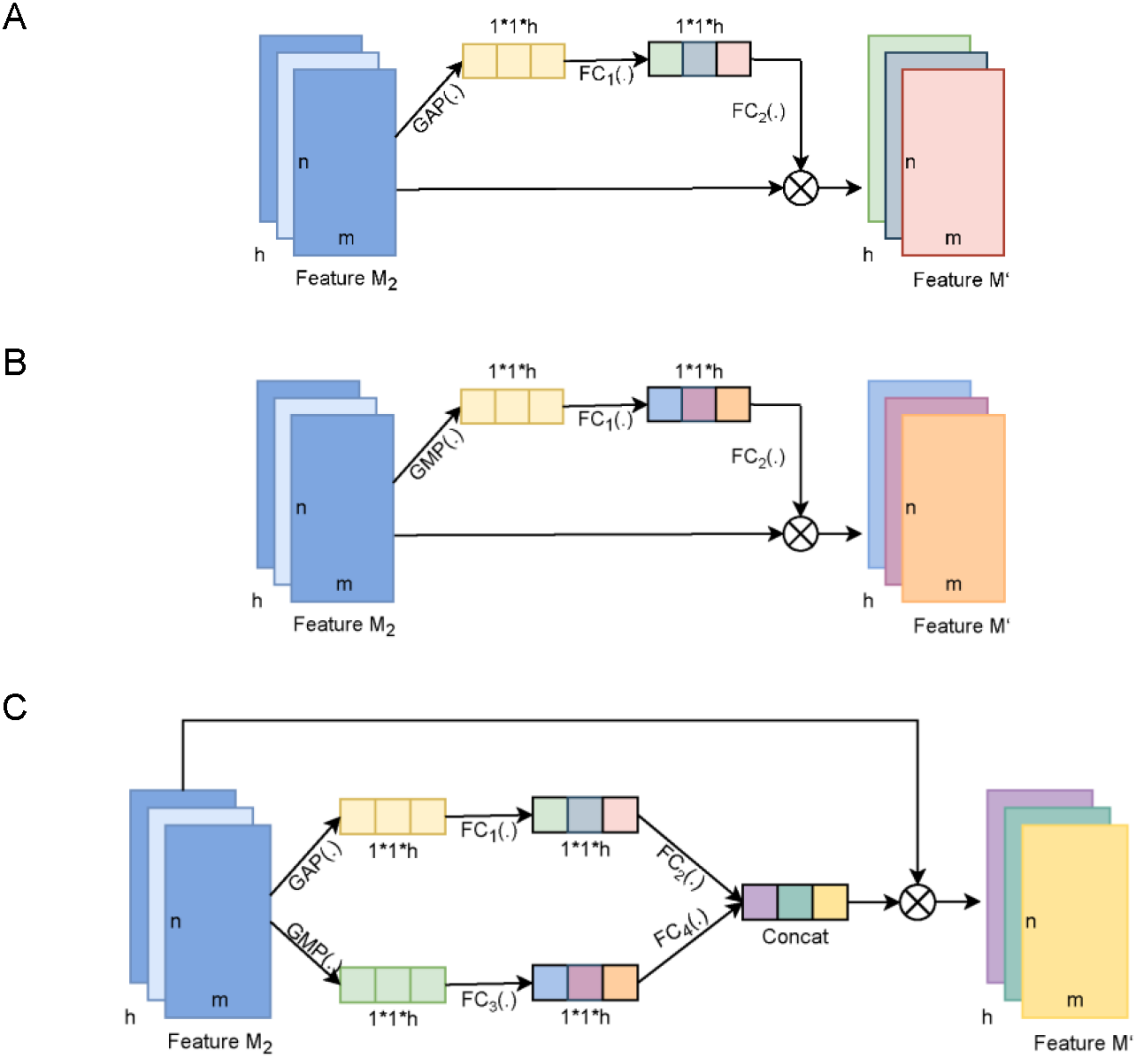
The diagram of the channel attention of MDCapsUbi and its variant structure. (A) The architecture of AvgNet. (B) The architecture of MaxNet. (C) The architecture of ConcatNet. Feature M_2_ and Feature M’ of dimension m*n*h in (A, B and C) represent the input map and output map respectively. The dimensions of the trainable weight features in (A, B and C) are 1*1*h. GAP(.) in (A and C) represents global average-pooling. GMP(.) in (B and C) represents global max-pooling. FC_1_(.), FC_2_(.), FC_3_(.) and FC_4_(.) in (A, B and C) represent fully connected networks.

The MDCapsUbi aggregates the information of each feature map through the global average-pooling operation and further trains the weight features corresponding to different feature maps to achieve feature recognition in the feature map dimension. We can call this channel attention mechanism. Different channel attention structures extract different features of data. There are several related attention methods (Fig. 4). We compared and discussed the attention mechanism of MDCapsUbi with related methods.

AvgNet (Fig. 4A) compresses feature maps by global average-pooling and aggregates background information of feature images. MaxNet (Fig. 4B) differs from AvgNet in that MaxNet uses the global max-pooling operation to compress feature maps and aggregates texture information of feature maps. ConcatNet (Fig. 4C) uses both global average-pooling and global max-pooling to aggregate the background information and texture information of the input feature map *M*_2_. The aggregated features of the two types of information are then fed into the respective fully connected networks for training to obtain the weight features, and finally the attentional feature map incorporating background and texture information is obtained by element summation.

As shown in Table 2, AvgNet achieved the best performance, followed by ConcatNet and MaxNet. It could be concluded that background information of feature maps was more important than texture information for protein ubiquitination data. In the identification of protein ubiquitinated sites, we speculated that the texture information obtained by global max-pooling could only represent part of the interaction between amino acids on the protein sequence, while the background information obtained by global average pooling was the aggregation of complex interaction between different amino acids.

**Table 2:** Performance comparison of the three channel attention structures.

Method	Acc	Sn	Sp	MCC	F-Score	AUC
MaxNet	0.8954	0.9090	0.8818	0.791	0.897	0.94
AvgNet	0.9182	0.9139	0.9224	0.837	0.919	0.97
ConcatNet	0.9017	0.8922	0.9111	0.804	0.900	0.96

### Performance of MDCapsUbi on single species data

The “equivariant” of capsules enables the model to achieve good performance on small data sets (Wang, Liang & Xu, 2019). To further validate the performance of MDCapsUbi on small data sets of single-species, the data of 60,879 ubiquitinated sites were further divided according to different species. Seven single-species datasets with the number of positive data between 200 and 20,000 were obtained and shown in Table 3. The seven species included *Arabidopsis thaliana*, *Emericella nidulans*, *Mus musculus*, *Toxoplasma gondii*, *Oryza sativa*, *Saccharomyces cerevisiae* and *Rattus norvegicus*. To avoid the imbalance of positive and negative samples, this module also randomly selected negative samples with the same number of positive samples. We selected 10% of the data from each species dataset separately as independent test sets, and the remaining 90% of the samples were used to train.

**Table 3:** Statistical summary of positive data sets constructed for seven species.

Species	Number of positive data
*Arabidopsis thaliana*	2171
*Emericella nidulans*	3245
*Mus musculus*	4782
*Toxoplasma gondii*	668
*Oryza sativa*	376
*Saccharomyces cerevisiae*	5367
*Rattus norvegicus*	885

**Figure 5:**
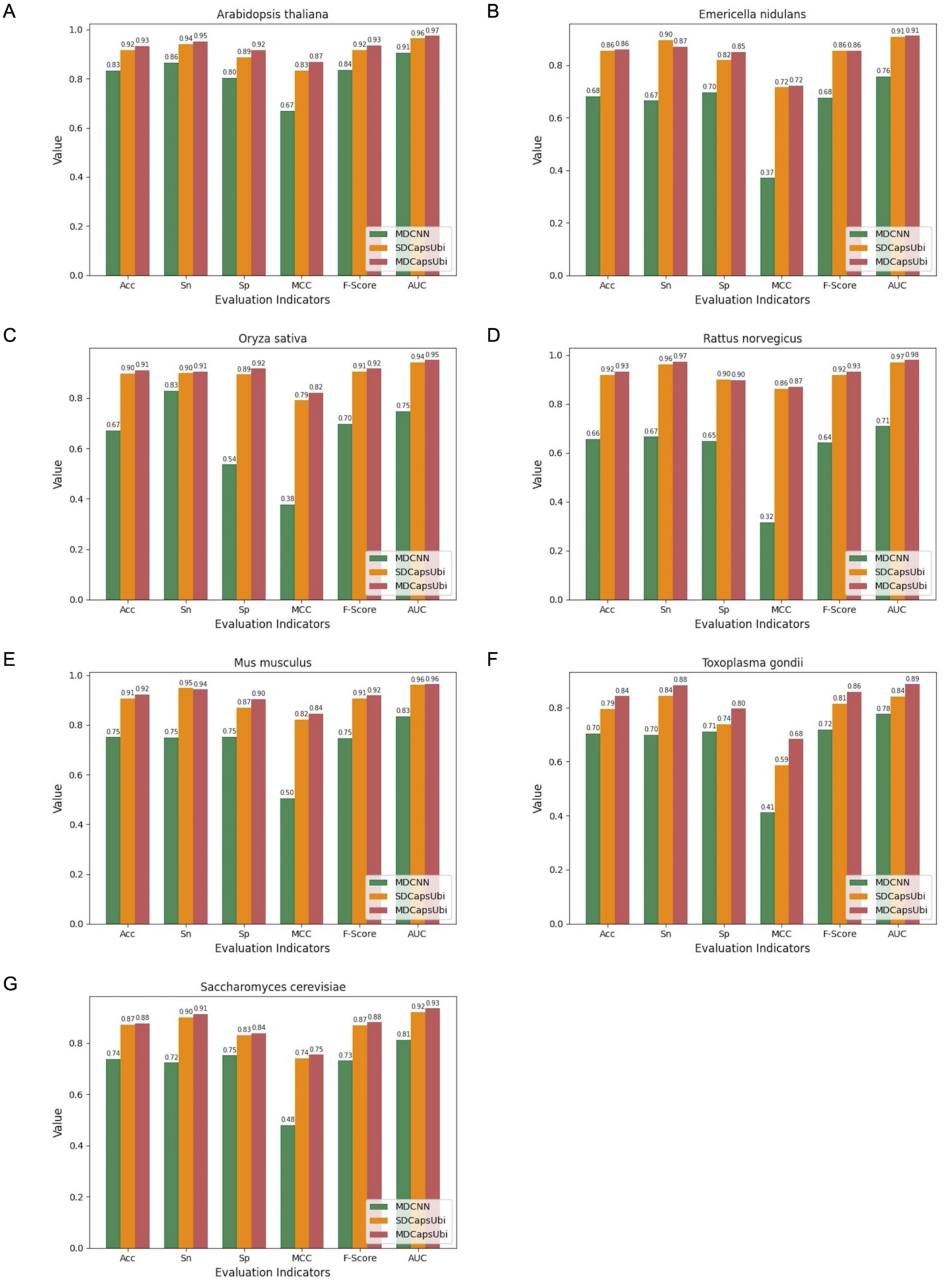
Performance comparison of MDCapsUbi, SDCapsUbi and MDCNN on single-species data sets. (A) The performance in *Arabidopsis thaliana*. (B) The performance in *Emericella nidulans*. (C) The performance in *Oryza sativa*. (D) The performance in *Rattus norvegicus*. (E) The performance in *Mus musculus*. (F) The performance in *Toxoplasma gondii*. (G) The performance in *Saccharomyces cerevisiae*.

We applied the data of the seven species to MDCNN, SDCapsUbi and MDCapsUbi respectively. MDCNN model which had a similar structure and complexity to MDCapsUbi consisted of SEM, MD-FRM, and a convolutional neural network module. Each model was cross-validated and tested by independent test sets. Finally, the test results were averaged and drawn into bar charts (Fig. 5). The experimental results revealed several advantages of MDCapsUbi: (1) The overall performance of MDCapsUbi consistently outperformed SDCapsUbi and MDCNN with single-species data. (2) Compared with MDCNN, MDCapsUbi showed greater performance improvements with small data sets compared to the models trained under mixed data. In *Arabidopsis thaliana*, MDCapsUbi increased accuracy by 10%, sensitivity by 9%, specificity by 12%, MCC by 0.2, *F*-Score by 0.9, and AUC value by 0.06 compared with MDCNN. This showed the more significant advantages of the capsule network compared to the CNN on small data sets. (3) By comparing the performance of MDCapsUbi and SDCapsUbi with the results, we could conclude that feature recognition in the feature map dimension played a greater role in single-species ubiquitination site prediction. (4) The model performance of *Arabidopsis thaliana*, *Emericella nidulans*, *Oryza sativa* and *Rattus norvegicus* was generally higher than that of the mixed data, while the model performance of *Mus musculus*, *Toxoplasma gondii* and *Saccharomyces cerevisiae* was lower than that of the mixed data. This may be due to the fact that the motifs detected by models of the latter three species were not significant enough compared with the others.

### Interpretability of MDCapsUbi

**Figure 6:**
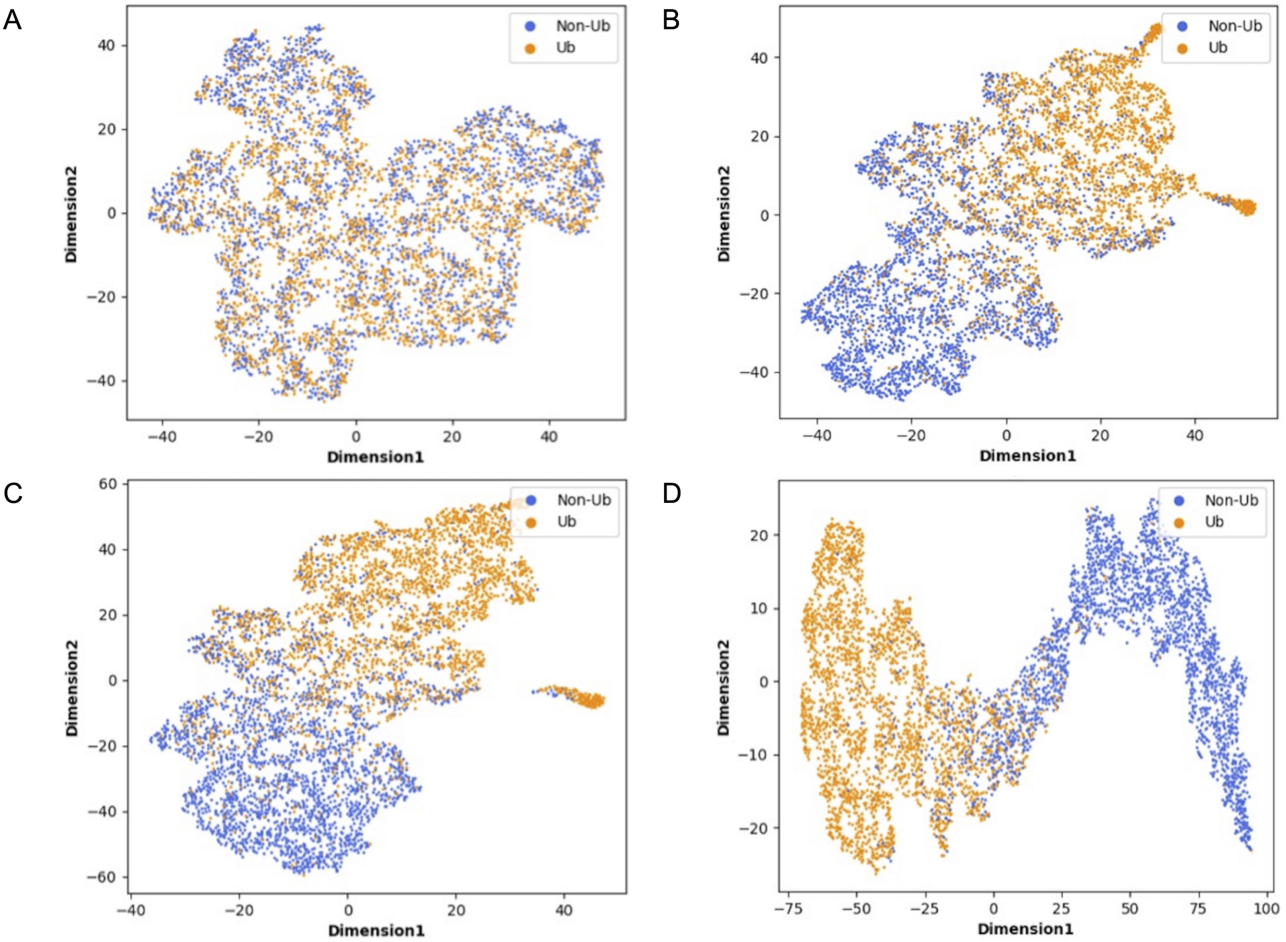
Visualization of each MDCapsUbi module using t-SNE. (A) Representation of raw data. (B) Representation of positive and negative samples after identifying the features of the sequence dimension. (C) Representation of positive and negative samples after identifying the features of the feature map dimension. (D) Representation of positive and negative samples after classification by capsule network.

Although deep learning models have achieved great research results in many fields, their internal structures are complex. Deep learning models have been criticized for their black box effect caused by high nonlinearity (Guo et al., 2020). Therefore, it is challenging to improve the interpretability of the model. To present the role of each module of MDCapsUbi more intuitively, t-SNE (Van Der Maaten, 2014) was used to visualize each module during testing. The t-SNE converts the similarity between data into probability and maps the data from the high-dimensional space to the low-dimensional space, while it still preserves the local characteristics of the dataset. We used t-SNE on the independent test set and visualized the features extracted from each module as a two-dimensional scatter diagram. We used different color scatters to distinguish positive and negative samples, where orange points represented ubiquitinated sites and blue points represented non-ubiquitinated sites. The positive and negative samples of the raw input data were completely mixed (Fig. 6A). Then, the separation trend of positive samples and negative samples was becoming more and more obvious (Figs. 6B–6D). After the initial feature identification in the sequence dimension by the convolution operations, there was an initial trend of separation between positive and negative samples. Channel attention further identified features in the feature map dimension, and the separation of positive and negative samples was more obvious. Finally, the capsule network refined the features and realized the classification of ubiquitinated sites, and the scatter points of positive and negative samples were easily separable.

**Figure 7:**
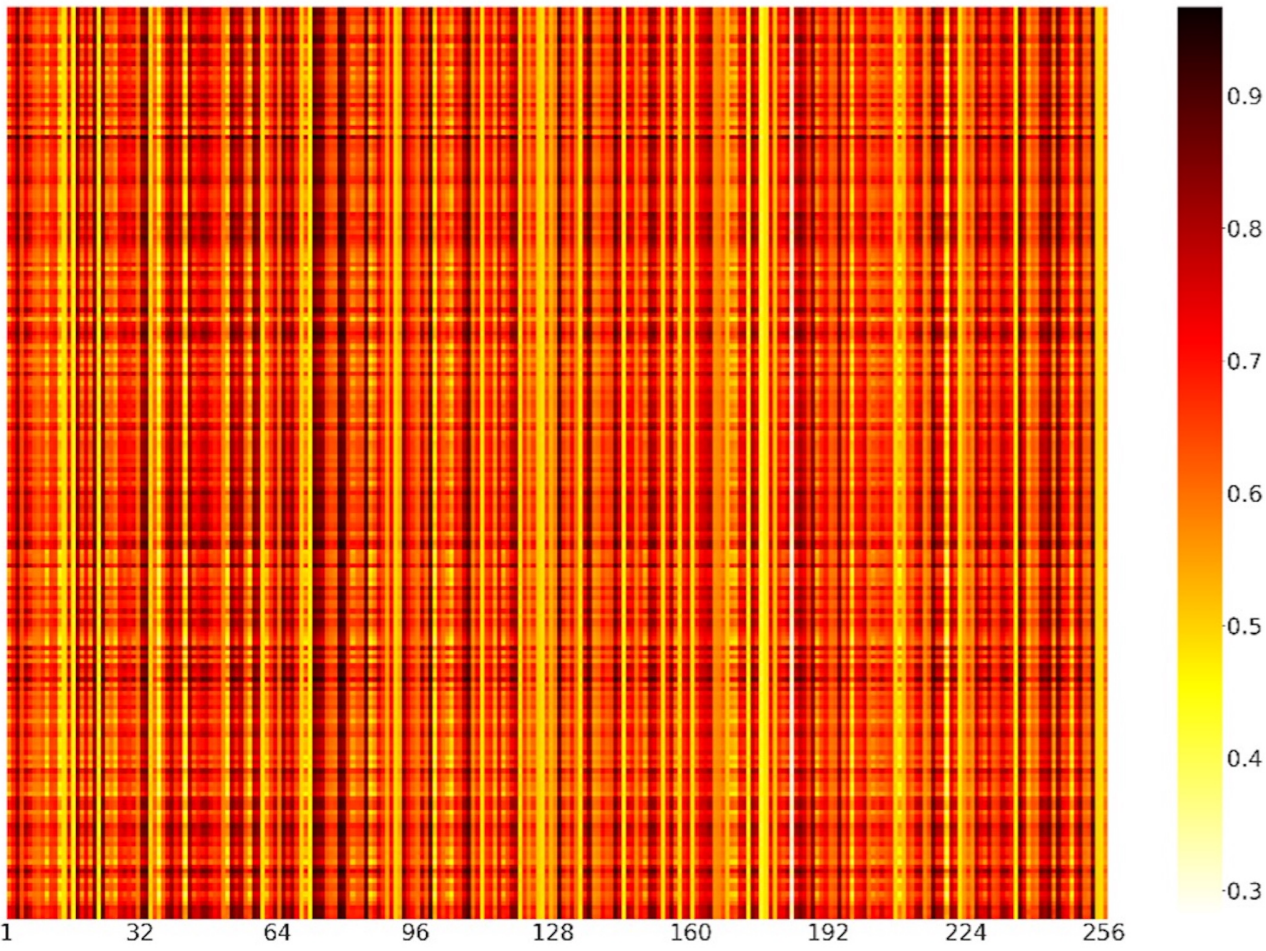
Heatmap visualization of channel weights. The abscissa represented the position of the channels, and the ordinate represented the position of the positive samples. Each element represented the value of the channels’ weight.

To further explain what was learned in the model of channel attention and demonstrate the role of feature recognition in the feature map dimension, we randomly selected 200 positive samples from the independent test set. The weights of positive samples in each channel of the channel attention module were shown in the form of a heatmap (Fig. 7). Each element represented the value of the channels’ weight. The darker the color of the channel, the more important the channel was. We could find that the colors of channels 33, 72, 73, 78, 79, 84, 99, 108, 129 and 253 were relatively darker, so the feature maps corresponding to these channels had more influence on the model. In contrast, channels 14, 16, 71, 111, 144, 173, 176, 183, and 235 were relatively lighter in color, so the influence of the feature maps corresponding to these channels was relatively less for the model.

## Discussion

### Comparison of models on independent test sets with existing methods

ESA-UbiSite (Wang et al., 2017), UbiProber (Chen et al., 2013a; Chen et al., 2013b), iUbiq-Lys (Qiu et al., 2015) and Ubisite (Huang et al., 2016) are some of the popular ubiquitination site prediction tools that support bulk samples. UbiSitePred (Cui et al., 2019) is a novel machine learning model for predicting ubiquitination sites. And deepUbiquitylation (He et al., 2017) and DeepUbi (Fu et al., 2019) are two deep learning models. To prove the superiority of the model in practical application, MDCapsUbi was compared with the above prediction tools. Due to the same data set used for deepUbiquitylation, the results of deepUbiquitylation were directly referenced. We replicated UbiSitePred and DeepUbi based on the source code they provided, using the same training set and independent test set as MDCapsUbi. The comparison results are shown in Table 4.

**Table 4:** Comparison of MDCapsUbi with other prediction tools under independent test set.

Method	Acc	Sn	Sp	MCC	AUC
ESA-Ubisite	0.6126	0.4614	0.6334	0.064	–
UbiProber	0.5506	0.6240	0.5405	0.107	–
iUbiq-Lys	0.8463	0.3350	0.9688	0.005	–
Ubisite	0.7363	0.2962	0.7964	0.073	–
UbiSitePred	0.8234	0.8195	0.8274	0.647	0.91
deepUbiquitylation	0.6643	0.6667	0.6640	0.221	0.73
DeepUbi	0.8293	0.7297	0.9288	0.674	0.92
MDCapsUbi	0.9182	0.9139	0.9224	0.837	0.97

Compared with other models, MDCapsUbi achieved higher Acc, Sn, MCC and AUC values, and was superior to most models in Sp. The overall performance of the multi-dimensional feature recognition model based on capsule network outperformed the above comparison models. This indicates that MDCapsUbi has better performance on the protein ubiquitination site prediction problem.

The previous study (Wang et al., 2020) has shown that there are significant differences in protein sequence characteristics among animals, plants and fungi. Therefore, the prediction model is interfered by inter-species characteristic differences during training. To investigate the ability of MDCapsUbi to resist disturbance of feature differences between species, the dataset of Plant-UbiPred was used for MDCapsUbi. The trained model was called Plant-MDCapsUbi. The test results of the above model are shown in Table 5.

**Table 5:** Comparison of MDCapsUbi and Plant-UbiPred.

Method	Acc	Pre	Sn	F-Score	AUC
Plant-UbiPred	0.756	0.733	0.767	0.749	0.81
Plant-MDCapsUbi	0.892	0.865	0.936	0.898	0.95
MDCapsUbi	0.918	0.913	0.914	0.918	0.97

Compared with Plant-UbiPred, Plant-MDCapsUbi achieved better performance in plant ubiquitination site prediction. It shows that the multi-dimensional feature recognition based on capsule network can extract features more effectively compared with word-embedding scheme. In addition, MDCapsUbi is insensitive to the negative effects of characteristic differences between different species and has stronger robustness and generalization ability.

### Comparison of MDCapsUbi and convolutional neural network

**Figure 8:**
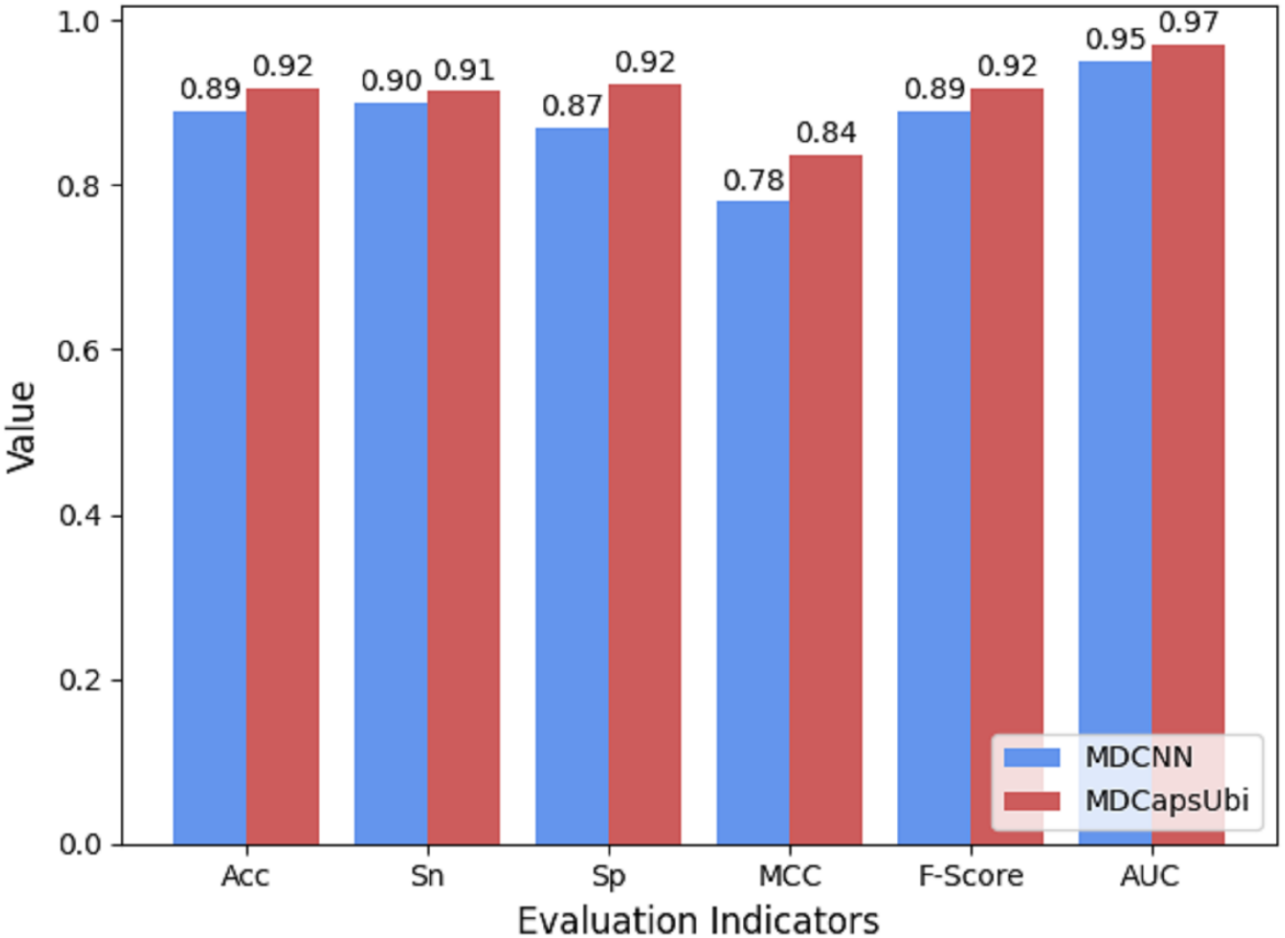
Performance comparison of MDCapsUbi and MDCNN.

The low-level capsules of the capsule network module are used to fuse and refine the features extracted by MD-FRM, and the activation of the low-level capsules to the high-level capsules is achieved through the dynamic routing mechanism. To show the superiority of capsule network compared to traditional convolutional neural networks, we compared the CapsNM with a CNN having the corresponding structure. The same protein encoding method and training strategy were used for MDCapsUbi and MDCNN, and ten-fold cross-validation was carried out in the same training set and test set. Compared with MDCNN, MDCapsUbi improved Acc by 3%, Sn by 1%, Sp by 6%, MCC by 0.06, F -Score by 0.03, and AUC by 0.02 (Fig. 8).

In addition to improving the performance of the predictor, developers should also explore the underlying biological significance of the model. CNN can be viewed as a feature detector, and similarly, so can capsule networks. MDCapsUbi’s capsules can be used to fuse and refine features. In biology, the features recognized by the capsules are the motifs of the protein sequences (Khanal et al., 2022; Wang, Liang & Xu, 2019). Motifs represent the ubiquitinated features of protein sequences, which often signal post-translational modification sites (Bulavka et al., 2021).

## Conclusions

In this article, we proposed a new prediction tool, MDCapsUbi, which was a deep learning model using capsule network for protein ubiquitination site prediction. MDCapsUbi consisted of three parts: SEM, MD-FRM and CapsNM. It took the original protein sequences as input data and identified coarse-grained features of sequence dimension and feature map dimension under the effect of convolution and channel attention. Then it obtained fine-grained features and further identified ubiquitinated sites through the capsule network. To recognize the dependencies of potential features between channels more effectively, we tried three channel attention methods: MaxNet, AvgNet and ConcatNet. Ultimately, we found that the channel attention of the AvgNet structure could better improve the feature representation of MDCapsUbi. MDCapsUbi could also be applied to the problem of single-species ubiquitination site prediction on small data sets. With the same dataset, MDCapsUbi had better performance compared to existing deep learning models. In addition, the model outperformed other popular machine learning models and MDCNN with similar structure. Experimental results showed that the effective combination of MD-FRM and CapsNM enabled MDCapsUbi to have better feature recognition ability on long-range sequences. We also visualized the attentional weights of channel attention in the form of a heatmap and visualized the classification effects of each module of the model using t-SNE.

Since there are characteristic differences among different species, it is necessary to conduct targeted studies on different species and further develop species-specific computational models based on capsule network. Second, the proposed model only takes protein sequences as input data, and the features identified by the model are also based on the features of the original protein sequences. Researchers can also try to combine other specific features of proteins, such as shape features, structural features, *etc.*, and further realize the combination of computational methods with biological mechanisms of protein ubiquitination. Although MDCapsUbi is currently applied only to the protein ubiquitination site prediction, we believe that it has great potential in other biological sequence-based identification and analysis studies.

## Additional Information and Declarations

### Data Availability

The following information was supplied regarding data availability: The data and code are available at GitHub: https://github.com/WeiMin-Li-visual/MDCapsUbi; WeiMin-Li-visual, & FangYu-Liu. (2022). WeiMin-Li-visual/MDCapsUbi: First release of MDCapsUbi (v1.0.0). Zenodo. https://doi.org/10.5281/zenodo.7275824.
